# Diagnostic value of molecular approach in screening for fragile X premutation cases

**DOI:** 10.1007/s11845-022-03166-9

**Published:** 2022-11-21

**Authors:** Miral M. Refeat, Mostafa M. El Saied, Ehab R. Abdel Raouf

**Affiliations:** 1 Medical Molecular Genetics Department, Human Genetics and Genome Research Institution, National Research Centre, Cairo, Egypt; 2 Department of Research On Children With Special Needs, Centre of Excellence of Medical Research, National Research Center, Cairo, Egypt

**Keywords:** Fragile X syndrome, Full mutation, Intermediate alleles, Methylation-specific PCR, mRNA (FMR1), Premutation alleles

## Abstract

**Background:**

Fragile X syndrome (FXS) is the most common form of inherited intellectual disability, caused by CGG-repeats expansion (> 200 repeats). Premutation alleles (PM) (55–200 CGG repeats) are associated with tremor ataxia syndrome (FXTAS), fragile X-associated primary ovarian insufficiency (FXPOI), and autistic problems.

**Aim:**

To screen the frequency of premutation carriers using molecular diagnostic assays, in a cohort of Egyptian males with suspected clinical features of (FXS) checking for the presence of premutation alleles.

**Methods:**

The current study comprised 192 Egyptian male children, 92 participants presented with intellectual disability, delayed language development, autistic-like features, behavioral difficulties, anxiety, seizures, and depression compared to 100 healthy males. All cases were subjected to clinical and neuroimaging assessments, when indicated as well as molecular analysis using methylation-specific PCR (MS-PCR) and quantitative real-time PCR (qRT-PCR).

**Results:**

Thirty-four premutation carriers out of 92 Egyptian males (37%) of CGG repeats (55 to 200) were illustrated with elevated FMR1 mRNA expression level (*p*-value < 0.001). Additionally, 2 intermediate (IM) cases (0.03%) (45–55 CGG repeats) showed poor increase in expression level (*p*-value = 0.02838) plus 6 full mutation (FM) patients (0.07%) with (> 200 CGG repeats) (*p*-value < 0.001) resulted in *FMR1* gene silence.

**Conclusion:**

Molecular diagnostic assay including (MS-PCR) and (qRT-PCR) proved to be a sensitive and rapid screening tool for the detection of premutation cases. Furthermore, the presence of positive correlation between FMR1 mRNA expression levels with CGG repeats in premutation cases could serve as a potential diagnostic marker. Application of these diagnostic tools on larger number clinically suspected cases is recommended.

## Introduction

Fragile X syndrome (FXS, OMIM #300,624) is one the most common cause of hereditary intellectual disability disorders with prevalence 1/4000 in males and 1/8000 in females. FXS is caused by CGG trinucleotide expansion for more than 200 triplets in the 5′ untranslated region of fragile X mental retardation gene 1 (*FMR1*) gene located at Xq27.3. Incidence of premutation (PM) alleles (55–200 CGG repeats) is 1/151 in females and 1/380 in male carriers. Meanwhile, the frequency of intermediate or gray zone alleles (45–54 CGG repeats) is 1/35 in females and 1/42 in males [1]. FXS patients suffer impaired synaptic plasticity and connectivity in the brain, which in turn leads to intellectual disability (ID) with an intelligence quotient (IQ < 70), autism spectrum as well as other clinical features including large head, elongated face, protruding ears, hyperextensible finger joints, flat feet, and large testes in postpubertal males [2]. Fragile X premutation allele is associated with Fragile X-associated tremor/ataxia syndrome (FXTAS) and fragile X-associated primary ovarian insufficiency (FXPOI) [3]. FXTAS is a late onset neurodegenerative condition that represents 40% of adult male premutation carriers. It is characterized by progressive development of intention tremor and ataxia often accompanied by progressive cognitive dysfunction as well as behavioral difficulties including anxiety, depression, and sometimes occurrence of seizures [4]. In spite of increasing recognition of defined clinical phenotypes in FM adults, there is still a shortage in awareness about the role of premutation alleles in development of neurodevelopmental problems in early childhood among those who are suspected to be carriers [5]. The gray zone or intermediate (IM) alleles do not demonstrate any of the clinical symptoms of full mutation FXS but it has been suggested to be more related to the premutation-FXTAS. The number of repeats can increase a little from one generation to another but does not proceed to a full mutation and the offsprings of carrier mothers are particularly at high risk of PM inheritance [6]. Fragile X mental retardation (*FMR1*) gene regulates the synthesis of fragile X mental retardation protein (FMRP), RNA-binding protein that plays an important role in regulating proliferation and differentiation of neural stem-progenitor cells. There is a significant association between the levels of FMR1 mRNA and CGG repeats size in children with FXS syndrome [7]*.* FXS full mutation alleles are caused by silencing of (*FMR1*) gene and the lack or deficiency of the FMR1 protein FMRP in males, affecting synaptic plasticity and connectivity in the developing brain leading to intellectual disability (ID) and other clinical features of FXS. Meanwhile, premutation alleles are associated with high expression levels of FMR1 mRNA; lead to calcium dysregulation, mitochondrial dysfunction, and destruction of proteins important for neuronal function; and enhances neuronal cell death. These features are associated with “mRNA gain of function” neuronal toxicity and result in disorders such as (FXTAS). Poor elevation of FMR1 mRNA expression levels might indicate the intermediate or gray zone alleles [8]. Different molecular genetic techniques have been set for diagnosing of FXS including conventional PCR, quantitative real-time PCR (qRT-PCR) but methylation-specific PCR (MS-PCR) assay is considered to be a rapid, high-throughput, easy to perform, cost-effective, sensitive, and specific [9]. Current medical treatments focused on symptoms and disorders linked to FXS, including ADHD, anxiety, and behavior disorders. Variety of different medications including drugs for management of attention deficit and hyperactivity are recommended for anxiety and associated disrupted behavior [10]. The aim of the current study is to screen for the presence of premutation alleles in a cohort of Egyptian males with suspected clinical features of FXS using MS-PCR and qRT-PCR as a rapid and sensitive tool to improve diagnostic efficiency and genetic counseling for FXS “FM and PM” cases and their families.

## Methods

### Participants

The current study comprised 192 Egyptian male children, 92 studied cases (age from 6 to 14 years; mean ± SD: 7.7521 ± 1.017) compared to 100 healthy controls, their age from 5 to 16 years (mean ± SD: 7.321 ± 1.075). Patients and healthy volunteers were subjected to detailed medical, family history, and specific neurological examination. Participants were recruited from Neuro-Rehabilitation and Learning Disabilities Clinic, Centre of Excellence of Medical Research, National Research Centre, Cairo, Egypt, during March 2020 till February 2022. An informed consent form was signed from the accompanying parents or guardian that was approved by the Medical Research Ethics Committee, NRC.

### Clinical examinations

Patients were subjected to clinical, neurological examinations, psychometric, and comprehensive evaluation. MRI was requested when needed. The inclusion criteria according to Hagerman’s checklists [11] were intellectual disability, autism spectrum, learning problems, seizures, hyperactivity, anxiety, obsessive compulsive symptoms, depression, migraines, developmental delay, physical abnormalities, cognitive impairment, and short attention span. Other clinical features included large prominent ears, hand-flapping; hyperextensibility of joints, and macroorchidism in post pubertal males. All participants had an intelligence quotient (IQ) test measured by Wechsler Intelligence Scale for Children Fifth Edition (WISC-V) [12]. Meanwhile, exclusion criteria included other causes of mental retardation like Down syndrome, syndromes with chromosomal anomalies, and severe neurological defects.

### Molecular analysis

Molecular analysis embraced three techniques: conventional PCR to amplify CGG repeats of the 5′ UTR and exon 1 of *FMR1* gene [13], bisulfite treatment followed by (MS-PCR) to demonstrate CGG repeats [2], and (qRT-PCR) using TaqMan assay to estimate expression levels of *FMR1* mRNA [14].

### PCR amplification of CGG repeats

#### Genomic DNA extraction

Genomic DNA was extracted from 200 μL peripheral blood lymphocytes of 100 participant (50 cases and 50 healthy controls) using Thermo Scientific Gene JET Genomic DNA Purification Kit (#K0721, Thermo Scientific, Waltham, MA, USA) according to the manufacturer’s instructions. Concentration and purity of DNA were quantified using nano drop and stored in aliquots at − 20 °C till analysis.

#### Conventional PCR

Amplification reaction of CGG repeats of the 5′ UTR and exon 1 of *FMR1* gene was carried out in 25ul using10 pmol fragile X forward and reverse primers that were designed according to genomic sequence (GenBank accession numbers ≠ NG_007529.1), 1 × buffer, 1 × Q-solution, 100 ng of genomic DNA, 200 uM from each of dATP, dCTP, dTTP, and 150 uM/50uM dGTP/7′ deaza-dGTP, 2U of Qiagen polymerase, and 1 × betain. PCR cycling conditions were generated in (PerkinElmer; USA) as follows: 99 °C for 10 min followed by 35 cycles of 99 °C for 1 min, 60 °C for 90 s, 75 °C for 2 min, and finally, extension at 75 °C for 10 min. PCR products were analyzed by 2% agarose, stained with ethidium bromide.

#### Methylation-sensitive PCR (MS-PCR)

Bisulfite treatment of DNA was done using EZ DNA Methylation-Gold Kit (Zymo Research, CA, USA) per manufacturer’s instructions. Two different MS-PCR reactions were performed in 25ul volume in (PerkinElmer; USA), each reaction contains 1 × buffer, 1 × Q-solution, 1 × betaine, and 200 uM from each of dATP, dCTP, and dTTP, together with 150 uM/50 uM dGTP/7′ deaza-dGTP, 2U of Qiagen polymerase, and 500 ng of the bisulfate-treated DNA. A total of 10 pmol of two different sets of primers, designed according to Methprimer (Thermofisher scientific, USA) software, was added in two separated reactions; one set was methylated primers designed for the PCR amplification of the methylated CpG island located upstream of the repeats of PCR cycles started with denaturation at 95 °C for 5 min, then 35 cycles at 95 °C for 1 min, 65 °C for 1 min, 75 °C for 2 min, and finally extension at 75 °C for 10 min. Meanwhile, the other set was unmethylated primer pairs for PCR amplification of fragments containing the unmethylated CGG repeats of thermal cycles proceeded with denaturation of PCR reaction at 95 °C for 5 min, followed by 35 cycles of 95 °C for 1 min, 58 °C for 1 min, and 75 °C for 2 min, and then a final extension 10 min at 75 °C. PCR fragments were analyzed by electrophoresis through 2% agarose gels stained with ethidium.

### Quantitative real-time PCR (qRT-PCR)

#### Blood samples collection

Total RNA was prepared from 3 ml of blood collected from each participant using RNA extraction kit (Qiagen, USA). Concentration of extracted RNA had been quantified using NanoDrop and stored in aliquots at − 20 °C. GAPDH (Thermo Scientific, USA) was used as an endogenous control for all samples to normalize the expression levels of target RNAs.

#### Reverse transcription of total RNA

Total RNA was reverse transcribed into cDNA using TaqMan RNA Reverse Transcription Kit (Applied Biosystem, USA) as manufacturer’s protocol. RT reactions were performed in 20 μL aliquots containing 500 ng of patient total RNA sample, 1 × RT buffer, 1.75 mM MgCl_2_, 0.5uM RT primers, 5 mM DTT, 0.5 mM dNTPs, 1.0 U/μL RNase inhibitor, and 2.5U reverse transcriptase. RT thermal cycling conditions were as follows: 25 °C for 10 min, 48 °C for 40 min, 95 °C for 5 min, and final cooling to 4 °C. RT reaction products were stored at − 20 °C.

#### Quantitative reverse transcription

Quantitative real-time polymerase chain reaction (qRT-PCR) assays of *FMR1* mRNA were performed using target-specific TaqMan Assays (Applied Biosystems, USA) according to manufacturer’s protocol. The reaction was composed of 100 ng of cDNA template, 1 × TaqMan Gene Expression assay, and 1 × TaqMan Gene Expression Master Mix, and then the volume was completed up to 20μL with RNase-free water. PCR cycling conditions were performed in 7500 step one real-time PCR system (Applied Biosystems, USA) as follows: 95 °C for 15 min and 50 cycles of 95 °C for 15 s and 60 °C for 1 min. Along with the Cq-values calculated automatically by the SDS software, raw data were exported for further analyses.

#### Statistical analysis

Relative quantification (Rq) of RNAs’ expression was calculated using the 2 − *ΔΔCT* method as 2 − (mean patient *ΔCt* − mean control *ΔCt*). *ΔCt* was verified by subtracting the *Ct* (threshold cycle) values for endogenous control GAPDH from the *Ct* values for the *FMR1* gene. Raw data were statistically analyzed using SPSS version 22.0 software (SPSS Inc., Chicago, IL, USA). *p*-value of less than 0.05 was considered statistically significant. Student *T* test was used to compare gene expression levels between groups, and correlations between gene expression levels. Clinical data was presented as the mean ± standard deviation (SD).

## Results

### Clinical results

The present study enrolled 92 Egyptian male children (age 6 to 14 years; mean ± SD: 7.7521 ± 1.017), presented with developmental delay, suggestive physical features of FXS, cognitive impairment compared to 100 healthy Egyptian males their age from 5 to 16 years (mean ± SD: 7.321 ± 1.075). Consanguinity in the studied families was 32%. Patients suffered intellectual disability (*n*: 86/92; 94%), autistic-like features (*n*: 68/92; 74%), attention-deficit-hyperactivity-disorder (ADHD) (*n*: 72/92; 78%), seizures (*n*: 37/92; 40%), hyperactivity (*n*: 60/92; 66%), anxiety (*n*: 66/92; 72%), physical abnormalities encompassed macrocephaly (*n*: 30/92; 33%), large oblong face with broad fore head and large jaw (*n*: 19/92; 21%), large prominent ears (*n*: 17/92; 19%), and hyperextensibile joints (*n*: 14/92; 15%) (Table 1). Intelligence quotient (IQ) test score of the 92 patients ranged from to 3585 with mean (73.26 ± 14.39). Thirty-four distinguished premutation alleles out of 92 patients (37%) were diagnosed with intellectual disability (*n* = 34/92; 37%) and autistic-like behavior (*n* = 34/92; 37%). Other associated clinical features were attention problems (*n* = 26/92; 28%), seizures (*n* = 32/92; 35%), hyperactivity (*n*: 29/92; 32%), anxiety (*n*: 31/92; 34%), sleep apnea (*n*: 23/92; 25%), depression (*n*: 24/92; 26%), migraines (*n*: 21/92; 23%), and obsessive compulsive symptoms (*n*: 25/92; 27%). Sixty out of 92 cases (65%) manifest of echolalia (Table 2). Individuals with IM-sized alleles do not demonstrate any of the clinical symptoms of full mutation FXS. It has been suggested that (IM) carries are at a high risk of later developing PM-related (FXTAS) and (FXPOI).

**Table 1:** Suspected clinical criteria of fragile X patients

**Clinical criteria**	**Number (%)**
Age	(6 to 14) (7.7521 ± 1.017)
Intellectual disability	(*n*: 86/92; 94%)
Autistic like behavior	(*n*: 68/92; 74%)
Attention-deficit-hyperactivity-disorder (ADHD)	(*n*: 72/92; 78%)
Seizures	(*n*: 37/92; 40%)
Anxiety	(*n*: 66/92; 72%)
Hyperactivity	(*n*: 60/92; 66%)
Physical abnormalities encompassed macrocephaly	(*n*: 30/92; 33%)
Large oblong face with broad fore head and jaw	(*n*: 19/92; 21%)
Large prominent ears	(*n*: 17/92; 19%)
Hyperextensibile joints	(*n*: 14/92; 15%)
Intelligence quotient (IQ) test score	85 down to 35 (73.26 ± 14.39)

*IQ Test score*, intelligence quotient intelligence quotient (IQ) test score, *(ADHD)* attention-deficit-hyperactivity-disorder

**Table 2:** Clinical features of detected premutation carriers

**Clinical criteria**	**Percentage (%)**
Intellectual disability	(*n* = 34/92; 37%)
Autistic like behavior	(*n* = 34/92; 37%)
Attention problems	(*n* = 26/92; 28%)
Seizures	(*n* = 32/92; 35%)
Hyperativity	(*n*: 29/92; 32%)
Anxiety	(*n*: 31/92; 34%)
Sleep apnea	(*n*: 23/92; 25%)
Depression	(*n*: 24/92; 26%)
Migraines	(*n*: 21/92; 23%)
Obsessive compulsive symptoms	(*n*: 25/92; 27%)
Individuals manifest of echolalia	(*n*: 60/92; 65%)

*IQ Test score*, intelligence quotient Intelligence quotient (IQ) test score, *PM* premutation

### Molecular results

#### Conventional PCR

**Fig. 1:**
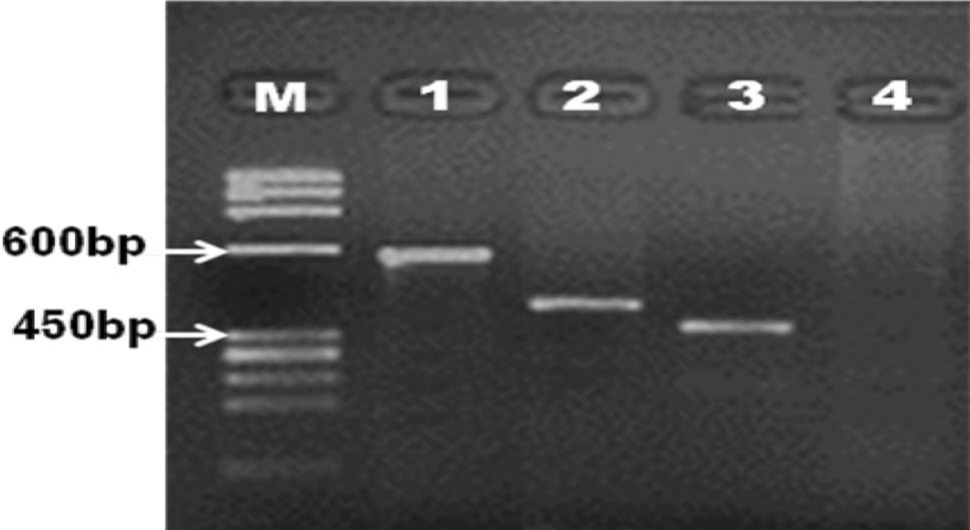
Agarose gel electrophoresis (2%) of amplified PCR products: M: DNA marker, lane 1, 2, 3, 4: PCR products of; premutation carriers (600 bp), intermediate (gray zone) cases (500 bp), normal individuals (450 bp), and full mutation patients (> 700 bp) (DNA smears of amplified that usually undetected on gel) respectively

Conventional PCR of CGG repeats of the 5′UTR and exon 1 of *FMR1* gene illustrated premutation alleles with CGG repeats ranged from 55 to 200 in 34 (37%) cases of PCR product 600 bp, 2 (0.03%) intermediate cases or gray zone alleles (45–55) of PCR product 500 bp, full mutations with CGG repeats of more than 200 in 6 (0.07%) patients of PCR product greater than 700 bp and 50 (63%) normal cases amid clinical features common with those of FXS, of PCR product 450 bp compared to control group that showed normal clinical and molecular picture (Fig. 1).

#### MS‑PCR

**Fig. 2:**
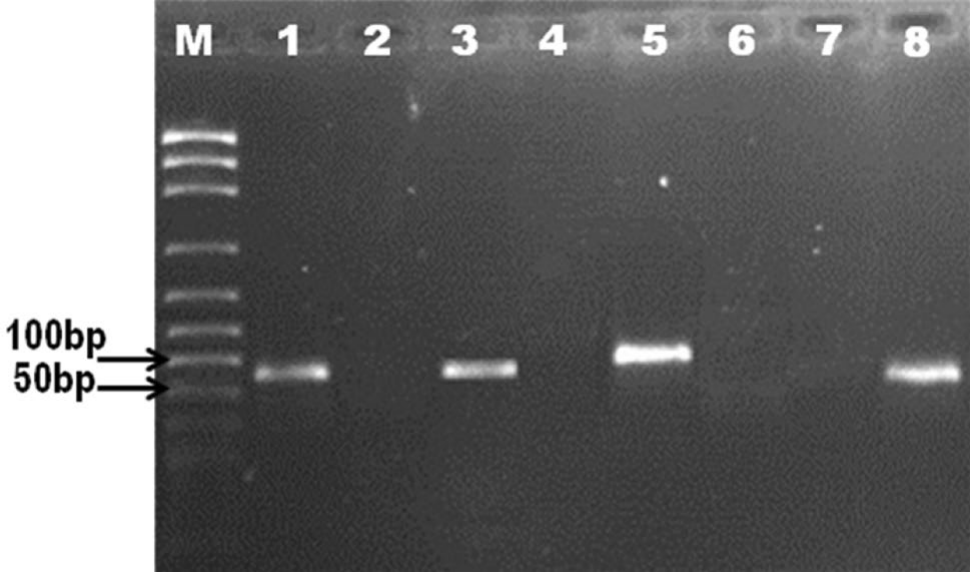
Agarose gel electrophoresis (2%) of amplified PCR products: M: DNA marker. Lane 1, 3, 5, 7: PCR product with unmethylated primers of: normal male (PCR product of 80 bp), intermediate cases (85 bp), premutation carriers (100 bp), and full mutation patients (no product). Lane 2, 4, 6, 8: PCR product with methylated primers of: normal male, intermediate cases, premutation carriers, and full mutation patients (PCR product of 80 bp) respectively

MS‑PCR amplification using unmethylated primers revealed PCR fragment of 100 bp in 34 premutation carriers and 85-bp fragment in 2 intermediate (gray zone) cases in addition to 80-bp fragment in 8 normal males but no PCR product detected in 6 patients with full mutations. However, MS‑PCR amplification using methylated primers demonstrated amplified products of 80-bp fragments in 6 patients presenting full mutations and no PCR product shown in premutation carriers, intermediate (gray zone) individuals, and normal males (Fig. 2).

#### Statistical results of RNA expression level

**Fig. 3:**
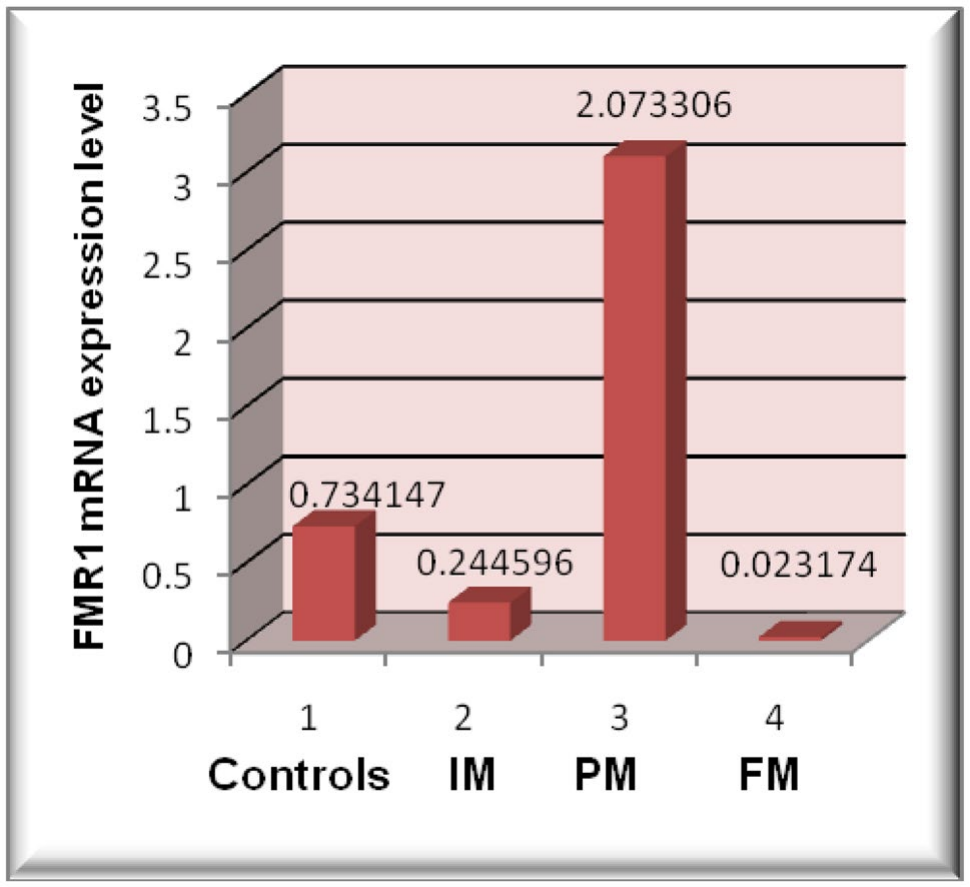
Relationship of controls, intermediate (gray zone) (IM), premutation (PM) and full mutations cases with FMR1 mRNA expression levels

**Fig. 4:**
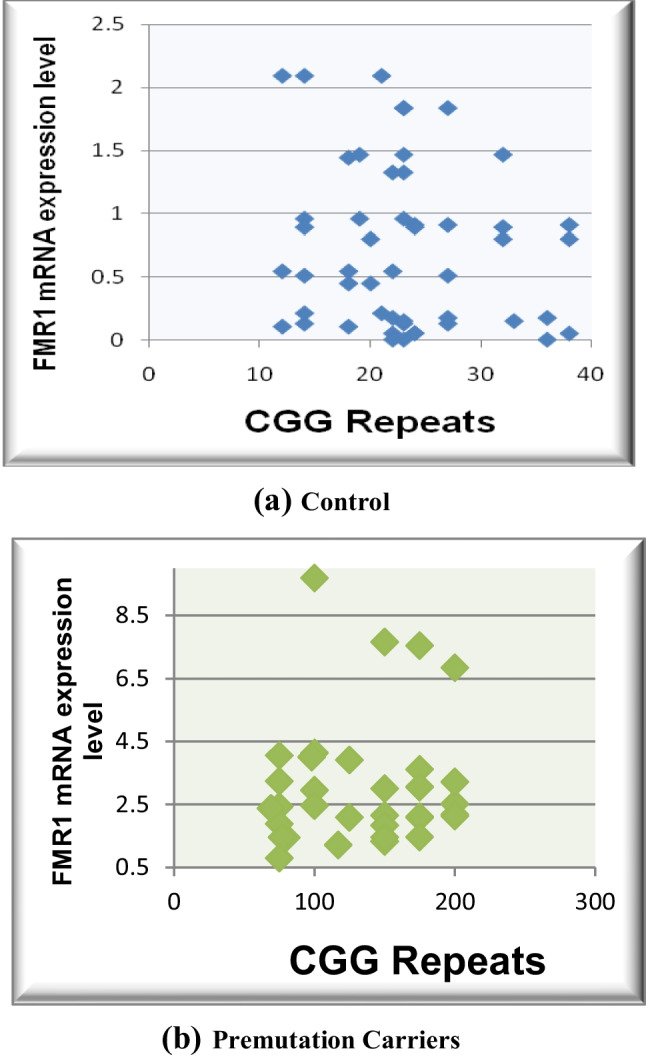
Statistical correlation of CGG repeats and FMR1 mRNA expression levels in controls (**a**) and in premutation carriers (**b**)

FMR1 mRNA expression levels and CGG expansion have been noticed to be correlated to IM, PM, and FM cases. FMR1 mRNA expression level was significantly high (*p*-value < 0.001) in 34 premutation carriers (2.073306 ± 0.60361; 55–200 CGG repeats), with 1.9-fold change compared to control group. Meanwhile, it showed reduced elevation of FMR1 mRNA expression levels within the 2 intermediate cases (0.244596 ± 0.187502; 45–55 CGG repeat) of (*p*-value = 0.02838) compared to healthy males. However, in the 6 full mutation patients (0.023174 ± 0.0178) with CGG repeats of more than 200 compared (*p*-value < 0.001) to healthy group with fold change 0.8 resulting in *FMR1* gene silence. Correlation of IQ test and CGG repeats as well as FMR1 mRNA expression level and CGG repeats was significantly high within IM cases, PM carriers, and FM patients of (*p* value < 0.001) (Figs. 3 and 4) (Table 3).

**Table 3:** Statistical correlation of CGG repeats and FMR1 mRNA expression levels

**Type**	**CGG repeats**	**FMR1 mRNA expression level**	***p***-**value**	**Significance**
Controls (*n* = 100)	23.74 ± 7.451	0.734147 ± 0.6391	< 0.001	HS
Intermediate (gray zone) cases (*n* = 2)	50 ± 4.242641	0.244596 ± 0.187502	0.02838	HS
Premutation carrier (*n* = 34)	128.6765 ± 53.73710	2.073306 ± 0.60361	< 0.001	HS
Full mutation patients (*n* = 6)	> 200	0.023174 ± 0.0178	< 0.001	HS

*HS* high significance

## Discussion

Fragile X syndrome (FXS) is an inherited X-linked monogenic disorder located at (Xq27.3Ch), associated with intellectual disability (ID) and autistic-like features. FXS is caused by expansion of CGG repeats (> 200) in fragile X mental retardation 1 gene (*FMR1*) and though, leading to full mutation FXS which in turn results in silence of *FMR1* gene. FXS is affecting 1 in 4000 males and 1 in 8000 females; meanwhile, premutation alleles represent 1 to 380 in males, and between 1 and 150 in females [15]. Premutation alleles (55–200 CGG repeats) are associated with clinical manifestations including fragile X-associated tremor ataxia syndrome (FXTAS), which exists in (40%) of adult males. The European Fragile X Network (EFXN), in 2019, had a symposium on recent research on conditions which affect fragile X premutation carriers; they agreed to use the term fragile X-associated neuropsychiatric disorder (FXAND), including auto-immune conditions to describe all conditions that might be associated with premutation syndrome [16]. The present study enrolled 92 Egyptian male children age ranged from 6 to 14 years (mean ± SD; 7.7521 ± 1.017) manifesting various clinical features similar to those typical of FXS cases described in previous studies [17]. They suffered from intellectual disability (*n*: 86/92; 94%), autistic features (*n*: 68/92; 74%), attention-deficit-hyperactivity-disorder (ADHD) (*n*: 72/92; 78%), seizures (*n*: 37/92; 40%), hyperactivity (*n*: 60/92; 66%), anxiety (*n*: 66/92; 72%), physical abnormalities encompassed macrocephaly (*n*: 30/92; 33%), large oblong face with broad fore head and large jaw (*n*: 19/92; 21%), large prominent ears (*n*: 17/92; 19%), and hyperextensibile joints (*n*: 14/92; 15%). Thirty-four identified cases with premutation alleles out of 92 patients (37%) showed intellectual disability (*n* = 34/92; 37%) and autistic like behavior (*n* = 34/92; 37%). They displayed other clinical features as attention problems (*n* = 26/92; 28%), seizures (*n* = 32/92; 35%), hyperactivity (*n*: 29/92; 32%), anxiety (*n*: 31/92; 34%), sleep apnea (*n*: 23/92; 25%), depression (*n*: 24/92; 26%), migraines (*n*: 21/92; 23%), and obsessive compulsive symptoms (*n*: 25/92; 27%), and the remaining 60 out of 92 cases (65%) manifest with echolalia [18]. Interestingly enough, two intermediate or “gray zone” alleles (45–54 CGG repeats) were discovered within the current study; about 14% of intermediate alleles are unstable and are considered to be precursors of premutation cases when transmitted from mothers to their offspring. However, to date it is not known if they would expand to full mutations hence leading to clinical manifestation of FXS [19]. Correlation was reported to be presence between several molecular elements, including CGG-repeat size, methylation, expression levels of FMR1 mRNA, and clinical FMR1-related disorders; FXTAS and FXPOI [2]. PM carriers may let their whole family be at potential risk of the disease that could be transmitted from one generation to another. Cascade testing is necessary to be requested throughout the family tree either by the physician or by the geneticists for precise genetic counseling [8]. Different molecular techniques have been used for diagnosis of FXS disorders including conventional PCR of CGG repeats in the 5′UTR and exon 1 of *FMR1* gene in addition to quantitative real-time PCR (RT-PCR) of FMR1 mRNA expression level, but the most preferable technique is methylation specific-PCR (MS-PCR) since it is considered to be an accurate, extremely rapid, and economic tool to be used as routine screening. To our knowledge, the current study is a pilot one in the Middle East that combines the triple entities in a single large screening for PM carriers, and application of these molecular techniques may allow us to diagnose larger number of premutation allele cases in future studies [9]. Quantitative reverse transcription assays (qRT-PCR) estimate the FMR1 expression levels to evaluate the full clinical spectrum and resolve the dilemma of overlapped clinical features of PM carriers that could be common with FM cases as ADHD, ASD, depression, and anxiety. This molecular-based discrimination between the two sets of patients (FM and PM) could be of significance value in clinical trials designed to serve as an outcome measure for treatments based on restoring FMR1 expression [20]. Despite advances in the understanding of the molecular aspects of FXS, clinical trials are still under research, as biology and phenotyping of FXS are more complex and many sub-groups of FXS exist. Few studies have conducted in Egypt on different cohorts of FXS including FM and PM cases. A simplified screening checklist for FXS was carried out on Egyptian patients with age ranging from 4.2 to 19 years, pre-diagnosed with idiopathic mental retardation using methylation-sensitive PCR (MS-PCR) technique. This checklist described FXS patients with large prominent ears, hyperextensibility of joints, and macroorchidism in post pubertal males. However, children with the premutation alleles had cognitive impairments, learning problems, attention deficit, developmental delay, and autistic features. Their IQ test score ranged from 20 to 85 with mean 63.08 ± 10.61; meanwhile, IQ score of participants in our study ranged from 85 down to 35 with mean (73.26 ± 14.39) [21]. Another Egyptian study recommended both clinical checklist score and (MS-PCR) technique to explore clinical and molecular correlation for 50 male patients characterized by intellectual disability (ID) and other FXS clinical manifestations compared with 50 healthy age-matched individuals; IQ test score of patients ranged from 35 to 70 [22]. In the present study, expression level of FMR1 mRNA is noteworthy related to the number of CGG repeats. PM alleles (55–200 CGG repeats) showed a prominent increase in FMR1 mRNA with high significant (*p*-value < 0.001) in 34 premutation carriers with 1.9-fold change compared to control group. Meanwhile, it showed reduced elevation in FMR1 mRNA expression levels within the 2 intermediate cases (45–55 CGG repeat) of (*p*-value = 0.02838) compared to healthy males. However, in the 6 full mutation patients with over 200 CGG repeats (*p*-value < 0.001), transcriptional silencing of FMR1 mRNA occurred and led to loss of FMRP protein with fold change 0.8. Significance variation in status of expression levels among FM, PM, and IM alleles may let us to expect phenotype-genotype correlation. Elevated expression level of *FMR1* mRNA in PM carriers is linked to more severe irritability symptoms and maladaptive behaviors; on the other hand, *FMR1* mRNA of transcribed FM alleles has negative implications of behavioral effects in males [23]. Two previous studies investigated the correlation between clinical phenotypes (intellectual disability, anxiety, and obsessive compulsive symptoms) and molecular measures (FMR1 mRNA expression levels as indicator of methylation status in the CGG repeat size in the promoter region). They concluded that premutation carriers diagnosed with higher levels of obsessive compulsive symptoms, depression, and anxiety comprised significantly elevated FMR1 mRNA expression level (*p*-value < 0.001) which consequently may result in neuronal toxicity; these changes may lead to neurodevelopmental problems, including frequent autism-like features and learning problems as well as neurodegenerative diseases including fragile X-associated tremor/ataxia syndrome (FXTAS) [24, 25]. Another, study utilized neuroimaging and clinical measurements of FMRI motor task in premutation carriers and a group of healthy males as controls. Variation in FMR1 transcription is associated with synthesis of FMR1 protein that is needed for typical brain development. It also highlighted molecularly the correlation of CGG repeats expansion and expression of FMR1 mRNA in PM and FM cases, using MS-PCR and qRT-PCR. The study illustrated that premutation carrier of (55–200 CGG repeats) and with elevated level of FMR1 mRNA expression demonstrated significantly sequential versus random finger flapping [26]. In the recent studies of FXS, PM alleles and other fragile X-associated disorders have been evoluted significantly due to the phenotype and genotype complexity of FXS. It has been clear that disorders related to expansion of CGG repeats of FMR1 gene and expression level of FMR1 mRNA are associated with a wide array of clinical presentations [3].

## Conclusions

The current study might help to highlight the role of premutation alleles in development of learning disabilities, which may pass without diagnosis either due to atypical presentation or the use of inappropriate laboratory workup. Clinical checklist including intellectual disability, learning problems, autistic features, anxiety, obsessive compulsive symptoms, and depression should be verified for diagnoses of fragile X premutation patients. An efficient management plan is required for improving the health of the premutation carriers, including a thorough screening of psychiatric problems in the family. Accurate family history may help carriers to understand the heredity mode of the diseases FXTAS or FXPOI in the family. These clinical characteristics may open avenue helping in diagnoses of premutation syndrome in childhood using the specific triple molecular approach conventional PCR, (qRT-PCR) and (MS-PCR). In our study, the use of the combined compound of triple molecular entities highlighted their beneficial value in discriminating PM cases from FM cohort. We recommend multicentre studies recruiting large number of cases for better delineation of phenotype genotype correlation in premutation cases.
